# Low-Cost Pocket Fluorometer and Chemometric Tools for Green and Rapid Screening of Deoxynivalenol in Durum Wheat Bran

**DOI:** 10.3390/molecules28237808

**Published:** 2023-11-27

**Authors:** Leonardo Ciaccheri, Annalisa De Girolamo, Salvatore Cervellieri, Vincenzo Lippolis, Andrea Azelio Mencaglia, Michelangelo Pascale, Anna Grazia Mignani

**Affiliations:** 1 CNR—Istituto di Fisica Applicata “Nello Carrara” (IFAC), Via Madonna del Piano, 10, Sesto Fiorentino, 50019 Florence, Italy; a.mencaglia@ifac.cnr.it (A.A.M.); a.g.mignani@ifac.cnr.it (A.G.M.); 2 CNR—Istituto di Scienze delle Produzioni Alimentari (ISPA), Via G. Amendola, 122/O, 70126 Bari, Italy; salvatore.cervellieri@ispa.cnr.it (S.C.); vincenzo.lippolis@ispa.cnr.it (V.L.); 3 CNR—Istituto di Scienze dell’Alimentazione (ISA), Via Roma, 64, 83100 Avellino, Italy; michelangelo.pascale@cnr.it

**Keywords:** deoxynivalenol, DON, wheat bran, fluorescence, chemometrics

## Abstract

Cereal crops are frequently contaminated by deoxynivalenol (DON), a harmful type of mycotoxin produced by several *Fusarium* species fungi. The early detection of mycotoxin contamination is crucial for ensuring safety and quality of food and feed products, for preventing health risks and for avoiding economic losses because of product rejection or costly mycotoxin removal. A LED-based pocket-size fluorometer is presented that allows a rapid and low-cost screening of DON-contaminated durum wheat bran samples, without using chemicals or product handling. Forty-two samples with DON contamination in the 40–1650 µg/kg range were considered. A chemometric processing of spectroscopic data allowed distinguishing of samples based on their DON content using a cut-off level set at 400 µg/kg DON. Although much lower than the EU limit of 750 µg/kg for wheat bran, this cut-off limit was considered useful whether accepting the sample as safe or implying further inspection by means of more accurate but also more expensive standard analytical techniques. Chemometric data processing using Principal Component Analysis and Quadratic Discriminant Analysis demonstrated a classification rate of 79% in cross-validation. To the best of our knowledge, this is the first time that a pocket-size fluorometer was used for DON screening of wheat bran.

## 1. Introduction

Cereals are a nutritious and convenient food option for a balanced diet given the range of health benefits they offer. Indeed, most cereals are a low-fat and a low-calorie source of carbohydrates, are rich in fibers, vitamins, and minerals, and help in lowering the cholesterol level [1,2,3,4]. Durum wheat bran for direct human consumption is one of the most added components in high-fiber breakfast cereals, bread and baked goods. It is also assumed as a dietary supplement being rich in magnesium, phosphorus and zinc, helping the immune system; moreover, it has a low glycemic index, thus improving the regulation of blood sugar and preventing spikes in insulin [4,5].

However, cereal crops, such as wheat, maize, barley, oats, and rye, are frequently contaminated by deoxynivalenol (DON), also known as vomitoxin (IUPAC name: 3α,7α,15-Trihydroxy-12,13-epoxytrichothec-9-en-8-one). DON is a type-B trichothecene mycotoxin, produced as a secondary metabolite of several species of *Fusarium* fungi. Furthermore, levels of DON in wheat bran have been found to be up to three times the levels in unprocessed wheat [6,7,8]. This contamination represents a significant threat to human health, as the ingestion of DON-contaminated cereals can cause digestive problems, nausea, vomiting, diarrhea, and other abdominal pains. In severe cases, it can also lead to more serious health issues such as anemia, decreased white blood cell count, and impaired immune function [9,10,11]. To protect the health of consumers from exposure to DON through the consumption of cereals and cereal-based products, the European Commission has set maximum permitted levels of DON in these food products. In particular, the EU maximum limit for DON in wheat bran intended for direct human consumption has been set at 750 μg/kg [12].

Conventional analytical methods for determination of DON in cereals mainly imply sample preparation and the use of gas chromatography (GC) with electron-capture or mass spectrometric (MS) detection or liquid chromatography (LC) coupled with UV/DAD or MS detection. Although these methods show high accuracy and precision, they are destructive, expensive, time consuming and unsuitable for screening purposes [13,14,15,16]. Factors like promptness and low cost of analysis, minimal sample preparation and environmentally friendly methods are of paramount importance for rapidly responding to the demands of the market. Consequently, in the last decade, other methods have gained wide acceptance such as rapid analytical tools for the screening of DON in cereals. Among them, heterogeneous assays like enzyme-linked immunosorbent assay (ELISA) and lateral flow immunoassay (LFIA), and homogeneous assays like fluorescence polarization immunoassay (FPIA) have been applied to the rapid screening of DON in wheat and derived products, including brans [17,18,19,20]. Although these approaches require only minimal sample preparation in terms of homogenization and extraction steps, they are still relatively labor intense, suffer from cross-reactivity of the antibody and require basic laboratory skills and equipment [21]. On the other hand, other approaches based on the use of electronic nose (e-nose) and optical techniques have been proposed as tools for the screening of cereal samples for DON content in a fast and non-destructive way [22,23,24]. In the last decade, e-nose methods have been proposed to indirectly assess the content of DON in wheat, barley and wheat bran samples by detecting changes in the composition of volatile organic compounds produced by mycotoxigenic fungi during their growth and biochemical processes [25,26,27]. Moreover, a variety of optical methods has been proposed for the indirect analysis of mycotoxins and fungal contamination in cereals by assessing their appearance and biochemical composition [28,29,30,31]. These optical methods include the use of infrared spectroscopy in the near (NIR) and middle (MIR) range and in the visible range with DON as the most targeted mycotoxin in wheat, maize, barley, and oat [32,33,34,35,36,37,38,39,40,41]. In some cases, the NIR range was complemented by reflectance measurements in the ultraviolet and visible bands [42,43,44]. Furthermore, Fourier transform (FT) instrumentation, offering several advantages compared to the traditional dispersive infrared instruments, has been used for the discrimination of DON in wheat and wheat bran [45,46,47,48,49]. NIR spectroscopy has been used more frequently compared to MIR for the prediction of mycotoxins, while imaging techniques like hyperspectral or multispectral imaging have been mainly used for the identification of fungal contamination in cereals. Table 1 summarizes the spectroscopic techniques that have been used to detect the DON contamination in the various types of cereals. The type of detection carried out, whether quantitative or qualitative (i.e., by classes of contamination), is also mentioned.

Fluorescence spectroscopy is another popular method employed in food analyses which has been used in the last decades, enabling the analysis of large-volume data for the identification of sample types and geographical origin of food, as well as for the detection of harmful substances such as mycotoxins and for the quantification of functional components [50,51,52]. Fujita and co-workers pioneered the use of fluorescence spectroscopy for detecting DON in water solutions using a wavelength range of excitation/emission of 200–340 nm and 500–600 nm, respectively [53,54]. Similarly, with the same scheme of the excitation–emission matrix, Sugiyama et al. (2011) applied fluorescence spectroscopy to wheat samples artificially contaminated with DON [55].

**Table 1:** Summary of spectroscopic techniques used to detect DON contamination in various types of cereals and the type of detection, whether quantitative or qualitative (i.e., classification based on mycotoxin contamination with respect to a threshold limit).

Spectroscopic Platform	Sample	Detection	Sensitivity	Reference
Near-infrared	Wheat flour	Qualitative	Threshold: 450 μg/kg	[32]
Near-infrared	barley	Qualitative	Threshold: 1250 μg/kg	[33]
Near-infrared	Maize	Quantitative	Limit of detection: 200 μg/kg	[34]
Near-infrared	Whole wheat grain	Quantitative	Limit of detection: 230 μg/kg	[35]
Near-infrared	Wheat kernel	Quantitative	Limit of detection: 400 μg/kg	[37]
Near-infrared	Barley	Quantitative	Limit of detection: 300 μg/kg	[38]
Near-infrared	Ground durum wheat	Qualitative	Threshold: 1400 μg/kg	[45]
Near-infrared	Ground durum wheat	Qualitative	Threshold ≤ 1000 μg/kg 1000 μg/kg < Threshold ≤ 2500 μg/kg Threshold > 2500 μg/kg	[46]
Mid-infrared	Maize	Qualitative	Threshold: 1250 μg/kg	[39]
Mid-infrared	Maize	Qualitative	Threshold: 560 μg/kg	[48]
Infrared	Wheat flour	Quantitative	Limit of detection: 440 μg/kg	[41]
Infrared	Maize	Qualitative	Threshold: 1250 μg/kg	[49]
Near/mid infrared	Wheat bran	Qualitative	Threshold: 400 μg/kg	[47]
Visible/near infrared	Ground oats	Quantitative	Limit of detection: ~200 μg/kg	[44]
UV/visible/near infrared	Maize kernel	Quantitative	Limit of detection: 1500 μg/kg	[42]
UV/visible/near infrared	Ground wheat	Quantitative	Limit of detection: ~200 μg/kg	[43]
Fluorescence	Wheat flour	Quantitative	Limit of detection: ~2.4 mg/kg	[55]

These spectroscopic methods show attractive features such as easy operation, no consumables, affordable cost, rapidity, little or no sample preparation and have the capability for a high sample throughput. Furthermore, as spectroscopy measures the sample as it is, non-destructively, and without any chemicals, solvents, or other treatments, it is considered a “green” analytics tool [56,57,58]. Indeed, it helps in reducing the environmental impact of food production processes and makes a significant contribution to both sustainable food production and environmental protection efforts [12].

The aim of the present work was to describe a low-cost, non-destructive, “green” and rapid method combined with chemometrics, for the screening of durum wheat bran samples by means of a low-cost pocket-size fluorometer. The cut-off level for DON screening was set at 400 µg/kg DON and was lower than the EU legal limit of 750 µg/kg set for the bran intended for direct human consumption [12]. This value was considered a safety limit, useful to take a decision whether accepting the sample as safe or carrying out a further inspection by means of more accurate but also more expensive standard analytical techniques. So far, to the best of our knowledge, there has been no literature reporting DON detection by means of a low-cost pocket-size fluorometer like the device shown in this paper.

## 2. Results and Discussion

**Figure 1:**
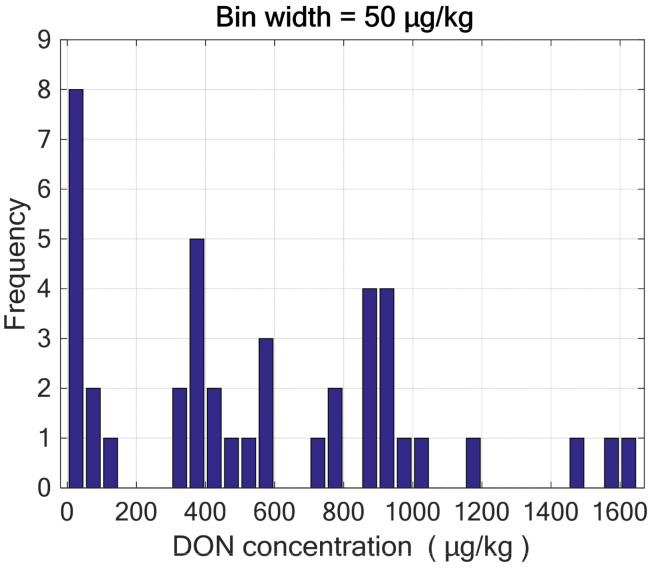
Distribution of DON content in the forty-two durum wheat bran samples as determined by the confirmatory method.

The DON level in durum wheat bran ranged from ≤40 µg/kg (limit of quantification of the HPLC confirmatory method) to 1650 µg/kg, as shown in Figure 1. Most samples (62%) were naturally contaminated with DON at levels lesser than the EU limit for wheat bran (750 µg/kg). These samples were grouped into two classes: 18 samples had a DON concentration equal to or lower than 400 µg/kg (Class A), while 24 samples had a DON concentration higher than 400 µg/kg (Class B).

**Figure 2:**
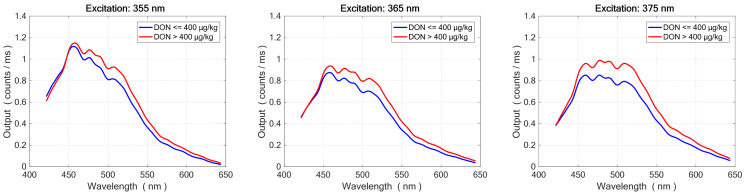
Examples of fluorescence spectra of DON-contaminated wheat bran samples belonging to Class A (≤400 µg/kg, blue line) or to Class B (>400 µg/kg, red line), excited at 355 nm (**left**), 365 nm (**center**), and 375 nm (**right**).

**Figure 3:**
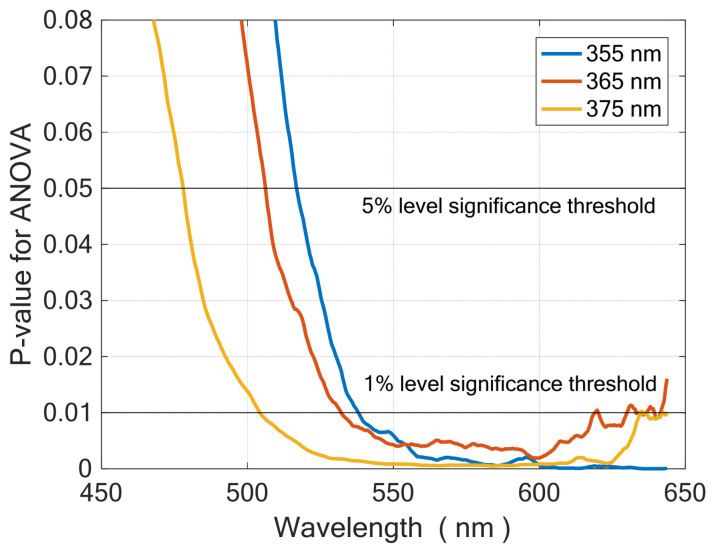
*p*-value of ANOVA as a function of emission wavelength for each excitation wavelength.

As an example, Figure 2 shows the average fluorescence spectra of wheat bran samples belonging to Classes A and B, excited at the three available wavelengths. An analysis of variance (ANOVA) was carried out at each wavelength to check the significance of difference between the two class means. The *p*-value, offering the probability that the difference between means is due to chance, was calculated. Figure 3 shows the *p*-value as a function of emission wavelength for each excitation wavelength. Significance threshold at a 5% level, which is commonly considered acceptable, as well as the 1% level threshold are also drawn in the plot. For each excitation wavelength, Table 2 summarizes the emission bands where the *p*-value is below a 1% or a 5% significance level threshold.

**Figure 4:**
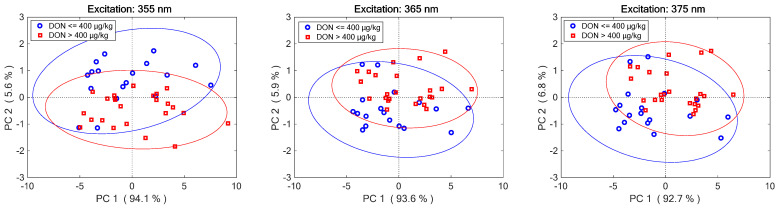
PCA score plots of training samples for the three excitation wavelengths (Ellipses represent 95% confidence limit of each class).

Although DON is not fluorescent [59], the occurrence of *Fusarium* fungi in the contaminated wheat bran slightly modified the fluorescence spectra of wheat by inducing different spectral shapes depending on the excitation wavelengths [60]. These effects were more than enough to clearly identify the two classes of contamination sought by using chemometric data processing. Figure 4 shows the Principal Component Analysis (PCA) score plots of training samples for the three excitation wavelengths. It is evident that each wavelength, taken alone, is only partially selective. Data fusion, however, combining information from all three models, achieved a better classification rate. All retained principal components were then merged into a predictor matrix that was fed into the classifier. For all wavelengths, two components were sufficient to explain 99% of total variance. This produced a total of six predictors, which were auto scaled (divided by their standard deviation) for assigning them equal *a priori* importance.

Table 3 summarizes the classification statistics for training and cross-validation, respectively, when considering a single excitation wavelength or combining the data of two or three excitation wavelengths. As described in Section 3.6, models were rated in terms of accuracy, sensitivity, and specificity. Accuracy is the overall classification rate, sensitivity is the true positive rate, and specificity is the true negative rate. The best single-wavelength models were those at 355 nm and 375 nm.

For each pair of excitation wavelengths, we calculated the correlations between the corresponding principal components. The components of the 365 nm model showed very strong correlation with those of both the 355 nm (PC1: 0.97, PC2: −0.97) and 375 nm (PC1: 0.97, PC2: −0.98) models. Instead, the components of 355 nm and 375 nm models showed weaker correlation (PC1: 0.91, PC2: −0.92). In fact, the 355 nm and 375 nm models excited with different efficiency the two fluorescent bands at 450 nm and 530 nm, as shown in Figure 2.

**Figure 5:**
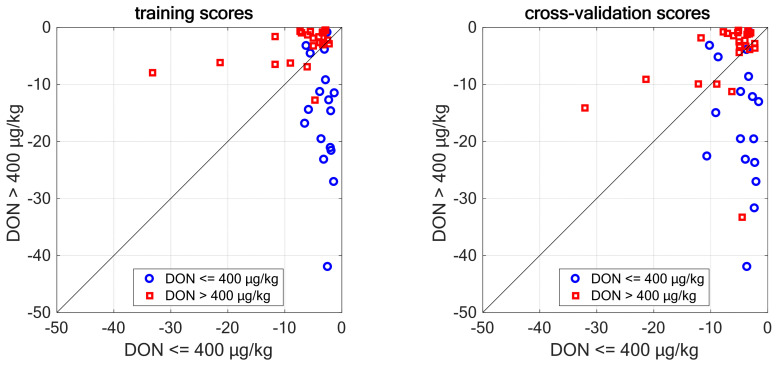
Quadratic Discriminant Analysis score plots for both training (**left**) and cross-validation (**right**) of the 355 nm + 375 nm model for classifying wheat bran samples contaminated with DON using intermediate-level data fusion.

The best two-wavelength model was achieved combining 355 nm and 375 nm, providing a 79% accuracy, a 75% sensitivity, and an 83% specificity in cross-validation. The three-wavelength configuration provided better results in training, but lower statistics in cross-validation, which are a 74% accuracy, a 71% sensitivity, and a 78% specificity in cross-validation. Clearly, adding 365 nm data increased collinearity in the model and introduced some overfitting. Figure 5 shows the plot of membership scores for the two best models. The probability for the lower class is measured along the *x*-axis, while that of the higher class is measured along the *y*-axis. The bisector of the first and second quadrant is the decision border.

For comparison with previous studies that reported the use of fluorescence spectroscopy for DON detection, we note that in those cases, the DON concentration was much higher, and that bulky and expensive bench-type fluorometers were used, having a wide excitation/emission matrix of 200–340 nm and 500–600 nm, respectively [53,54,55]. In particular, DON in water solution was measured from the 4 × 10^3^ μg/kg to 1 × 10^6^ range μg/kg [53], while DON in artificially spiked samples was measured from 2.4 × 10^2^ μg/kg to 26 × 10^3^ μg/kg [54,55].

## 3. Materials and Methods

### 3.1. Reagents and Apparatus

Acetonitrile of an HPLC grade was bought from Mallinckrodt Baker (Milan, Italy); ultrapure water was produced by a Millipore Milli-Q system (Millipore, Bedford, MA, USA). Deoxynivalenol (DON) standard was purchased from Sigma-Aldrich (Milan, Italy), while DON immunoaffinity columns (DONtest™ HPLC) were from Vicam, a Waters Business (Milford, MA, USA). Glass microfiber (Whatman GF/A) and paper filters (Whatman No. 4) were bought from Whatman (Maidstone, UK).

### 3.2. Durum Wheat Bran Samples

Forty-two samples (300 g each) of naturally contaminated durum wheat bran samples were collected from a local Italian mill farming. Each sample was finely ground by Tecator Cyclotec 1093 (International PBI, Hoganas, Sweden) laboratory mill equipped with a 500 μm sieve, leading to a homogeneous sample with a fine particle size. Then, samples were manually homogenized and stored at +5 °C until HPLC analysis and fluorescence measurements. 

### 3.3. Wheat Bran Sample Analysis by Reference Method

Milled durum wheat bran samples were analyzed by HPLC for the quantitative determination of DON to be used for the development of the fluorometric method. Each sample was analyzed according to the procedure described by De Girolamo et al. 2019 [47]. Briefly, 12.5 g of the ground sample were extracted by blending for 2 min with 100 mL of phosphate buffer solution (PBS, 10 mM sodium phosphate, 0.85% sodium chloride, pH = 7.4). Then, the extract was filtered through filter paper (Whatman No. 4) and then by glass microfiber filter (Whatman GF/A). Then, an aliquot of 2 mL of the filtered extract was cleaned up through the DONtest™ immunoaffinity column, and after washing the column by passing 5 mL of water through it, DON was recovered by elution with 1.5 mL of methanol. The eluate was dried under air stream at 50° C, redissolved into 0.25 mL of the mobile phase (acetonitrile/water, 8:92; *v*/*v*) and an aliquot of 0.05 mL was analyzed by LC (Agilent 1100 Series, Agilent Technology, Palo Alto, CA, USA) with the diode array detector set at 220 nm.

The DON levels in the 42 wheat bran samples ranged from ≥40 μg/kg (quantification limit of the HPLC method) to 1650 μg/kg.

### 3.4. Fluorometer Assembly

**Figure 6:**
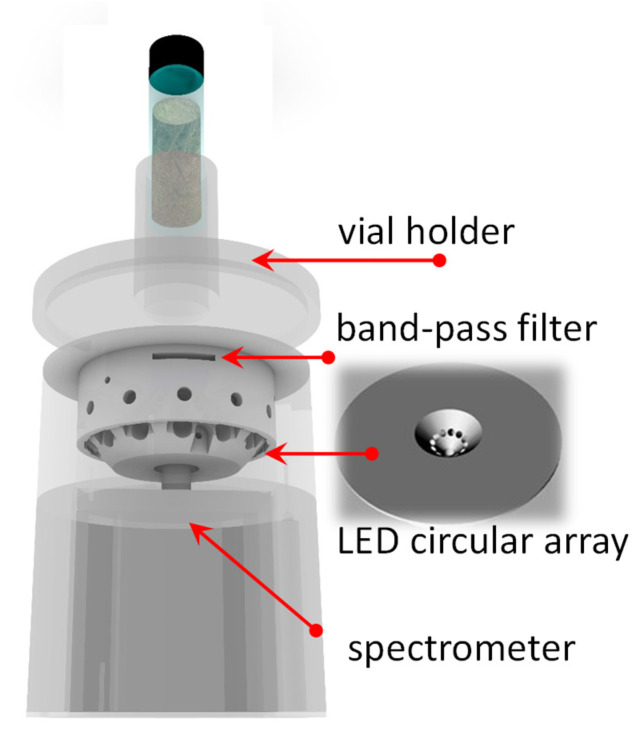
Rendering of the fluorometer.

The low-cost fluorometer built for this experiment was a pocket-size device that utilized an LED array for illumination and a miniaturized spectrometer for detection. The hardware module was operated by a laptop PC via a customized Labview^®^ software (Version 18.0.1f4) interface (National Instruments Corp., Austin, TX, USA). Figure 6 shows a rendering of the fluorometer. The optical head providing the fluorescence excitation was made of a housing for twelve circularly arranged LEDs. They were positioned at a 45° angle with respect to the axis of detection, which was in the center of the circle. Three wavelengths were considered for fluorescence excitation, i.e., 355 nm, 365 nm, and 375 nm. Four identical sets of these three LEDs were sequentially placed in circular array housing. They were powered by means of a commercially available electronic controller (PhidgetsLED1031, Phidgets Inc., Calgary, AB, Canada), which was USB-connected to a laptop PC. The optical head was butt-coupled to a cylinder housing the detector, which was an Ocean ST-visible microspectrometer (Ocean Insight, Duiven, The Netherlands) operating in the 350–810 nm range, having a 1.5 nm spectroscopic resolution. Because the LED spectrum extends in the visible range, a short-pass filter blocking illumination wavelengths longer than 400 nm was inserted between the optical head and the measurement spot. This filter had a small hole in correspondence of the spectrometer input slit to allow the detection of the backscattered fluorescence light intensity at wavelengths longer than 400 nm.

The three excitation wavelengths were operated in sequence (four LEDs at the same wavelength at a time), and the emitted fluorescence spectra were recorded synchronously with each excitation wavelength. Spectra acquisition was achieved using the USB port of the spectrometer, connected to the laptop. In practice, this scheme allowed us obtention of a low-cost, pocket-size, and versatile device providing a configuration similar to a three-wavelength scanning fluorescence spectroscopy.

A custom Labview^®^ (Version 18.0.1f4) interface was programmed for operating LEDs, spectrometer, detector acquisition, and the sequence of measurements. The software controlled the LED current, the acquisition from the spectrometer, and displayed the measured spectra that could be saved as an Excel file. Depending on the wavelengths, LEDs were powered with different currents for equalizing the light intensity used for fluorescence excitation. The custom software operating the fluorometer was programmed to acquire the “dark” background spectrum, according to the selected integration times, and to subtract it from the sample fluorescence spectrum.

**Figure 7:**
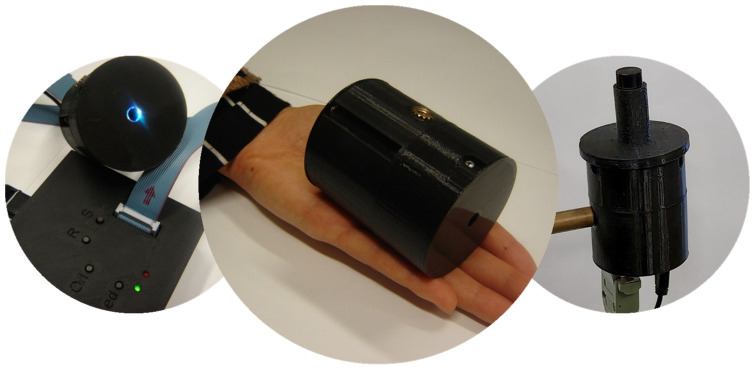
The fluorometer (**center**), with the supply unit (**left**) and with the vial holder (**right**).

The casing that housed all optoelectronic components was lightweight and handy. It was 3D printed (KLONER3D^®^240, KLONER3D^®^, Firenze, Italy) using a polylactic acid wire. A vial holder was also 3D printed to suitably position the bran sample in front of the illumination/detection optics. The practical implementation of the fluorometer including the vial holder is shown in Figure 7.

### 3.5. Wheat Bran Sample Analysis by Fluorescence

For each sample, an aliquot of 1.6 g wheat bran was transferred in a 4 mL glass vial sealed with a safety cap that was allocated in the vial holder and analyzed by the fluorometer. The fluorescence spectra of all bran samples, excited by the three wavelengths, were acquired by scanning 4 times each sample. The time analysis was approximately 13 s for all wavelengths.

### 3.6. Multivariate Statistical Analysis

Spectra processing and multivariate data analysis was carried out in MATLAB^®^ R2016b (MathWorks, Natick, MA, USA) using custom-made subroutines. For each sample, the average spectrum of the 4 scans obtained at each of the three wavelengths was used for data processing. Before development and validation of chemometric models, the fluorometric spectral data were preprocessed to reduce the spectral baseline shift and noise. A two-stage smoothing was applied to each spectrum. First, a 3 pts median filter was used for removing narrow noise spikes; then, a boxcar average filter with a 21 pts window (about 10 nm wide) was used for refined smoothing. Minimum subtraction was applied for removing the effect of random background fluctuations. As previously reported by De Girolamo et al. 2019 [47], a cut-off value of 400 μg/kg that was below the EC ML for DON in wheat bran (i.e., 750 μg/kg) [12] was used to discriminate wheat bran samples into two classes, A and B. Based on their DON content measured by HPLC analysis, samples were considered as compliant when [DON] ≤ 400 μg/kg (class A) and not compliant when [DON] > 400 μg/kg (class B). Using this screening approach, only samples classified as not compliant should be re-analyzed by the confirmatory method, thus reducing the total number of analyses.

The Leave-One-Out (LOO) cross-validation was used for evaluating the classification performance given the limited number of available samples.

Intermediate-level data fusion was employed for combining information coming from different excitation wavelengths. Spectral information was first compressed, for each excitation wavelength, by means of Principal Component Analysis (PCA) [61]. Then, significant principal components coming from all models were merged into a predictor matrix. Data fusion at PCA level allows reduction in the level of noise introduced in the model, because only structural information from each source is retained [62,63].

In each PCA model, two principal components explained 99% of total variance, offering a total of six predictors. Quadratic Discriminant Analysis (QDA) classification was applied to the joint predictor matrix for sample classification. Like Linear Discriminant Analysis (LDA), QDA assumes that all classes are distributed according to multivariate Gaussian distribution, but it does not assume all classes have the same within-class variance. It is, therefore, more flexible [64]. QDA assigned two scores to each sample depending on its position in the predictor space and proportional to the logarithm of class membership probabilities. Consequently, each sample was assigned to its most probable class. Test samples were projected onto each PCA model and then classified in the same way. Classification rates and confusion matrices were calculated for both training and test set for evaluating classifier performance. 

By assuming that Class A is that of non-compliant samples, samples were defined as true positive (TP) if they were correctly classified as A or false negative (FN) if they are classified as belonging to Class B. Similarly, samples of Class B considered compliant were defined as true negative (TN) if they were correctly found as belonging to Class B or false positive (FP) if they were classified as Class A. QDA performances on each set were assessed in terms of accuracy, sensitivity, and specificity according to the confusion matrices for binary classification [65,66] by using the following formulas:

Sensitivity = [TP/(TP + FN)] × 100,(1)

Specificity = [TN/(TN + FP)] × 100,(2)

Accuracy = [(TP + TN)/(TP + TN + FP + FN)] × 100,(3)

where

- sensitivity is defined as the fraction of samples belonging to Class A, correctly classified by the model and is a measure of the confidence level of the class space;
- specificity is defined as the fraction of samples not belonging to Class A that are correctly rejected by the model;
- accuracy is defined as the fraction of correctly classified samples with respect to the entire set.

## 4. Conclusions and Future Perspectives

An LED-based pocket-size fluorometer was developed in combination with chemometrics for green and rapid screening of DON in durum wheat bran samples. A cut-off limit of 400 µg/kg was set as a threshold useful to make a decision of whether to accept the sample as compliant with respect to the EU ML for DON in wheat bran or to carry out a further analysis by a more expensive standard analytical technique. 

By considering all excitation wavelengths, we spotted the wavelength combinations providing the best classification rates. The chemometric approach using intermediate-level data fusion (PCA + QDA) achieved classification rates in the 74–79% range in cross-validation, 355 nm + 375 nm being the best wavelength combination.

To the best of our knowledge, this is the first time that fluorescence spectroscopy carried out by means of a pocket-size fluorometer was successfully used for screening wheat bran naturally contaminated by DON with a contamination threshold of 400 μg/kg. Further activities will focus on making the fluorometer fully standalone by replacing the laptop PC currently used by means of a suitably programmed data processing unit and display. In addition, a new design of the cone-shaped optical head ending in a glass window is underway, so that the bran sample can be measured directly without using a vial for high-throughput screening. The ultimate objective is to transform this device into a high-throughput analytical platform that is cost effective for small mills. This pocket-size device will facilitate a rapid and non-destructive assessment of DON contamination onset during storage and pre-packaging stages without the need for chemicals or specialized by personnel, consequently ensuring the safety of the product before it enters the food chain.

## Figures and Tables

**Table 2:** Emission bands where the *p*-value is below a 1% or a 5% significance level threshold.

Excitation Wavelength	Significance at 5%	Significance at 1%
355 nm	above 517 nm	above 538 nm
365 nm	above 506 nm	532–619 nm
375 nm	above 478 nm	504–634 nm

**Table 3:** Performance parameters (accuracy, sensitivity, and specificity) of the quadratic discriminant analysis for both training and cross-validation, for the different excitation wavelengths and their data fusion. A cut-off of 400 μg/kg DON was used to distinguish the two classes of DON-contaminated wheat bran samples.

Wavelength (nm)	Training			Cross-Validation		
	Accuracy	Sensitivity	Specificity	Accuracy	Sensitivity	Specificity
355	81%	83%	78%	74%	75%	72%
365	71%	71%	72%	69%	67%	72%
375	74%	75%	72%	74%	75%	72%
355 + 365	83%	83%	83%	76%	75%	78%
365 + 375	86%	88%	83%	79%	83%	72%
355 + 375	86%	88%	83%	79%	75%	83%
355 + 365 + 375	88%	88%	89%	74%	71%	78%

## Data Availability

Data are contained within the article.
